# Use of latent class analysis as a method of assessing the physical activity level, sedentary behavior and nutritional habit in the adolescents’ lifestyle: A scoping review

**DOI:** 10.1371/journal.pone.0256069

**Published:** 2021-08-19

**Authors:** Valter Paulo Neves Miranda, Danilo Reis Coimbra, Ronaldo Rocha Bastos, Márcio Vidigal Miranda Júnior, Paulo Roberto dos Santos Amorim

**Affiliations:** 1 Department of Physical Education, Federal University of Viçosa, Minas Gerais, Brazil; 2 Department of Sports Science and Clinic Hospital (EBSERH), Federal University of Triângulo Mineiro, Uberaba, Minas Gerais, Brazil; 3 Department of Physical Education, Federal University of Juiz de Fora / Campus Governador Valadares, Governador Valadares, Minas Gerais, Brazil; 4 Department of Statistics, Geo-Referenced Information Lab (LINGE), Federal University of Juiz de Fora, Juiz de Fora, Minas Gerais, Brazil; 5 School of Physical Education, Physiotherapy and Occupational Therapy, Federal University of Minas Gerais, Belo Horizonte, Minas Gerais, Brazil; 6 Department of Physical Education, Federal University of Viçosa, Viçosa, Minas Gerais, Brazil; Universidade Federal dos Vales do Jequitinhonha e Mucuri, BRAZIL

## Abstract

**Background:**

Currently, adolescents’ lifestyle is commonly characterized by physical inactivity, sedentary behavior, and inappropriate eating habits in general. A person-oriented approach as Latent Class Analysis (LCA) can offer more insight than a variable-centered approach when investigating lifestyle practices, habits, and behaviors of adolescent population.

**Objective:**

The aim of the present study was to assess which variables are mostly used to represent the physical activity level, sedentary behavior SB) and nutritional habit in the adolescents’ lifestyle in studies that used the LCA.

**Design:**

Scoping review.

**Methods:**

The study was a performed in accordance with the proposed criteria for systematic reviews and meta-analyses—Preferred Reporting Items for Systematic Reviews and Meta-Analyses and registered in PROSPERO (CRD42018108444). The original articles were searched in MEDLINE/PubMed, Scopus, ScienceDirect, Web of Science, PsycINFO, and SPORTdiscus. The Quality Assessment Tool analyzed the risk of bias of the included studies.

**Results:**

30 original articles were selected. The physical activity level (28 studies), SB and nutritional habits (18 studies) were the most common variable used to evaluate the adolescent’s lifestyle by LCA model. Specifically, physical inactivity and high SB were the manifest variables with higher frequency in the negative latent classes (LCs) in adolescent girls. On the other hand, physical exercises and sports were activities more commonly labeled as positive LCs.

**Conclusions:**

The LCA models of the most of selected studies showed that physical inactivity, high SB were the most common in the LCs with negative characteristics of the adolescents’ lifestyle. Better understanding the results of analyzes of clusters of multivariate behaviors such as the LCA can help to create more effective strategies that can make the lifestyle of adolescents healthier.

## Introduction

Currently, adolescents’ lifestyle is commonly characterized by physical inactivity, sedentary behavior, and inappropriate eating habits in general [1]. Scientific studies have increasingly shown that adolescents do not practice the minimum recommendation of 60 minutes of moderate-to-vigorous physical activity (MVPA) daily [2]. In addition, this population spends almost one-third of their awake time (not sleeping) on sedentary behavior and eats large amounts of high-calorie, multi-processed foods and a low amount of fibrous vegetables [3, 4].

Recent research studies evaluating adolescent’s lifestyle behaviors have shown that the level of physical inactivity increases considerably over the lifespan, especially among girls [5–7] and older adolescents [8]. This increase is worrisome as the energy imbalances between the level of physical activity, sedentary behavior, and food intake are related to unhealthy weight development. These unhealthy behaviors and habits can decrease the cardiorespiratory fitness and as consequence to result in pediatric obesity [9], body fat accumulation and clinical manifestations of cardiometabolic diseases, inflammatory status [10]. Besides that, psychosocial consequences, such as low self-esteem, body image disturbance [11], eating disorder risk [12] and signs of depression and anxiety [13].

Recent studies have pointed out that adolescent’s lifestyle-change intervention programs need to include a multi-component lifestyle approach promoting physical activity and encouraging a healthy diet and other behaviors to effectively reduce overweight risk and excess of adiposity [14]. Thus, we highlight the importance of multivariate epidemiological studies to evaluate the relationship between different types of behaviors in adolescent health.

Mixture models have become widely used in behavioral sciences because they allow for an exploration of identification and understanding of latent subpopulations [15]. For example, latent class analysis (LCA), which is a subset of the structural equation modeling, is a precise and sophisticated way of clustering that can be used in the assessment of lifestyle practices based on the investigation of behaviors commonly adopted by adolescents [16, 17]. A person-oriented approach can offer more insight than a variable-centered approach when investigating lifestyle practices, habits, and behaviors of a specific population [18]. Response patterns can then be observed based on specific characteristics and related to a set of latent classes (LCs) [16].

The LCs are observed indirectly, and this approach is more focused on the features of individuals, which may be homogeneous or heterogeneous depending on the actual data structure [18, 19]. The LCs do not impose a predefined concept of that which is being observed [16]. For example, in a study developed by Miranda et al., LCA was used to evaluate the interaction between MVPA, number of steps, self-reported screen time, total sitting time, and number of meals and alcohol consumption as the covariates. The authors argued and discussed if the LCA was an accurate and objective method for the assessment of adolescent girls [20].

For a more accurate lifestyle assessment, the variables that represent behavioral aspects, such as physical activity level, sedentary behavior, food pattern, sexual practices, and the use of licit and illicit drugs, should be jointly analyzed by more robust statistical methods [21]. The problem is that most of the time, these behavioral variables in quantitative form hardly present a normal distribution or do not meet the assumptions necessary for the analyses. In contrast, LCA is appropriate for assessing the association of different kinds of categorical behavioral variables, which are common in lifestyle assessments.

Thus, we can consider the importance of LCA for public health because with this method information regarding the behavior and adolescent’s lifestyle from empirical studies. The multivariate analysis of different variables that are being used to create fit the LCA model, as such as, physical activity level (PAL), sedentary behavior (SB), dieting or eating habits, alcohol consumption, tobacco use, and other behaviors behavioral indicators can be important in developing strategies and interventions that will improve the health and development of adolescents. Therefore, this study aimed to undertake a systematic review to evaluate the characteristics of manifest variables and LCs of original studies that used LCA to assess the adolescents’ lifestyle and nutritional status.

## Material and methods

### Protocol registration

The study can be classified as a Scoping review, performed in accordance with the proposed criteria for systematic reviews and meta-analyses (Preferred Reporting Items for Systematic Reviews and Meta-Analyses) [22]. This Scoping review was used to map the concepts underpinning a research area and the main sources and types of evidence available [23]. In the case of the present study, the characteristics of the variables that were used in the studies that used the LCA to assess the adolescent’s lifestyle.

Our research was registered in the International Prospective Register of Systematic Reviews—National Institute for Health Research as CRD42018108444. The PRISMA checklist was made available as Supporting Information 1 (S1 Table).

### Search strategy

The MEDLINE/PubMed, Scopus, ScienceDirect, PsycINFO, SPORTdiscus, and Web of Science databases were systematically searched on February 5^th^ of 2021. Additional records were obtained from the references of the articles reviewed for eligibility. For each database, we used specific descriptors and keywords associated with the Boolean operators. The search terms were “AND” and “OR”: (“latent class analysis” OR “latent class growth analysis” OR “mixture modeling” OR “cluster patterns”) AND (“lifestyle” OR “lifestyle patterns”) AND (“adolescents” OR “children”).

There was also a search in the gray literature, mainly on Google Scholar. The search strategy was not limited by study design, language, or year. We intended to include all languages of dissemination but had to limit to English due to the large number of identified papers. In the MEDLINE and Scopus databases, filters were used to specify the search for studies that used the LCA to assess the adolescents’ lifestyle. Lastly, we also scanned references of a relevant review [23].

### Eligibility criteria

We searched original articles with cross-sectional, longitudinal, cohort, or experimental designs published in indexed peer-reviewed journals throughout the years until the end of the search period (February 2021).

In general, the adopted inclusion criteria looked for complete articles made available by the journal or the authors, and that used LCA as the primary method of analysis to evaluate the adolescent population (10 to 19 years old) lifestyles for both sexes. Lifestyles were understood as the behavioral variables related to the level of physical activity, sedentary behaviors, eating habits, abusive consumption of alcohol, and the use of tobacco and licit and illicit drugs. If the sample age range included children (inferior limit under nine years), but the mean age was inside the adolescent age, or if the author did not report the range or mean age, but classified them as adolescents, the study was included.

Duplicates, review articles, editorials, and letters to the editor were excluded; articles that were not fully available or did not have adolescents as the primary sample of the study were also excluded. In addition, we did not accept articles that did not use the behavioral variables as a basis for lifestyle assessment or used another statistical method of mixture models, for example, latent transitional analysis.

After using the eligibility criteria for the selection of records, the title and abstract of potentially relevant articles were analyzed. We reviewed the full article if the information regarding the lifestyle assessment of adolescents through LCA was not part of the title or the abstract.

The participants, interventions, comparisons, outcomes, and study design (PICOS) criteria used to define the research question were presented in Table 1.

**Table 1:** Participants, interventions, comparisons, outcomes, and study design (PICOS) used as eligibility characteristics in this systematic review.

PICOS criteria		Inclusion Criteria	Exclusion Criteria
**P**	Participants	Adolescent population (10 to 19 years old) lifestyles for both sexes	Children (0 to 9 years old), Adults (20 to 59 years old), and elderlies (≥60 years old).
**I**	Interventions	-	-
**C**	Comparasions	-	-
**O**	Outcomes	Lifestyles were understood as the behavioral variables related to the level of physical activity, sedentary behaviors, eating habits, abusive consumption of alcohol, and the use of tobacco and licit and illicit drugs.	Duplicates, review articles, editorials, and letters to the editor were excluded; articles that were not fully available or did not have adolescents as the primary sample of the study were also excluded.
		After using the eligibility criteria for the selection of records, the title and abstract of potentially relevant articles were analyzed.	If the article that assessed the adolescents’ lifestyle but did not use the ACL to assess the latent variable, this document was excluded.
**S**	Study desing	Original articles with cross-sectional, longitudinal, cohort, or experimental designs published in indexed peer-reviewed journals throughout the years until the end of the search period (May 2019)	Critical or Systematic reviews.

### Risk of bias assessment

The Risk of Bias was assessment of the included studies was analyzed through the Quality Assessment Tool [24], adapted for cross-sectional studies. This tool comprises eight components, including selection bias, study design, confounding factors, blinding, data collection methods, withdrawals, intervention integrity, and analyses. Each component was applied to the included studies which were then classified according to the general evaluation of the study. Thus, each article selected for analysis was considered ‘Strong Quality’, if it had no component classified as weak. ‘Moderate Quality’, if there was only one component classified as ‘Weak’. Finally, ‘Weak Quality’, if two or more components had a poor rating.

The processes of search, selection, and risk of bias were conducted by two members of the study team (VPNM and DRC). In case of disagreements, a third member (RRB) was consulted for a final opinion. One author (MVMJ) assisted in the selection process and discussion of the results.

### Analysis plan

For a more detailed analysis of the articles, we sought to identify the authorship, sample characterization, study and analysis characteristics, description and prevalence of the LCs generated by the model, number and type of manifest variables, odds ratio values of studies that included covariates, risk of bias, main conclusion about the LCA model, and evaluation criteria for the model.

In the selected articles, four highlight points were investigated to demonstrate the main characteristics of the LCA model that were generated from the assessment of lifestyle-related variables of adolescents:

1. Check if physical activity (PA) level, sedentary behavior (SB), nutritional or dietary habits, alcohol consumption, tobacco use, and other behaviors were used as manifest variables to create the LCA model.
2. Analysis of the characteristics of latent classes and the values of membership prevalence (γ). Posteriorly, the LCs generated by the models were classified according to the labels they received, based on the item-response probabilities for each category of the manifest variable. Thus, Negative LCs represent classes with unhealthy behaviors; Positive LCs represent classes with healthy behaviors, and Mixed LC represents classes with both healthy and unhealthy behaviors.
3. Verify which variables were used as covariates in the LCA model.
4. Besides that, analyses the odds ratio (OR) values that show the association these covariates have with the membership prevalence of the LCs.
5. Analysis of model selection and adjustment criteria: including tests of absolute model fit, assessment of relative fit of competing models, parsimony, interpretability, and degree of uncertainty (entropy).

## Results

The database search resulted in a total of 2,789 articles, with 286 records from MEDLINE/PubMed, 361 from Scopus, 840 from ScienceDirect, 617 Web of Science, 70 from PsycINFO, and 615 from SPORTdiscus. Eighteen articles were added after reviewing the list of references of articles analyzed for eligibility. Thus, a total of 2,807 studies were initially identified.

**Fig 1:**
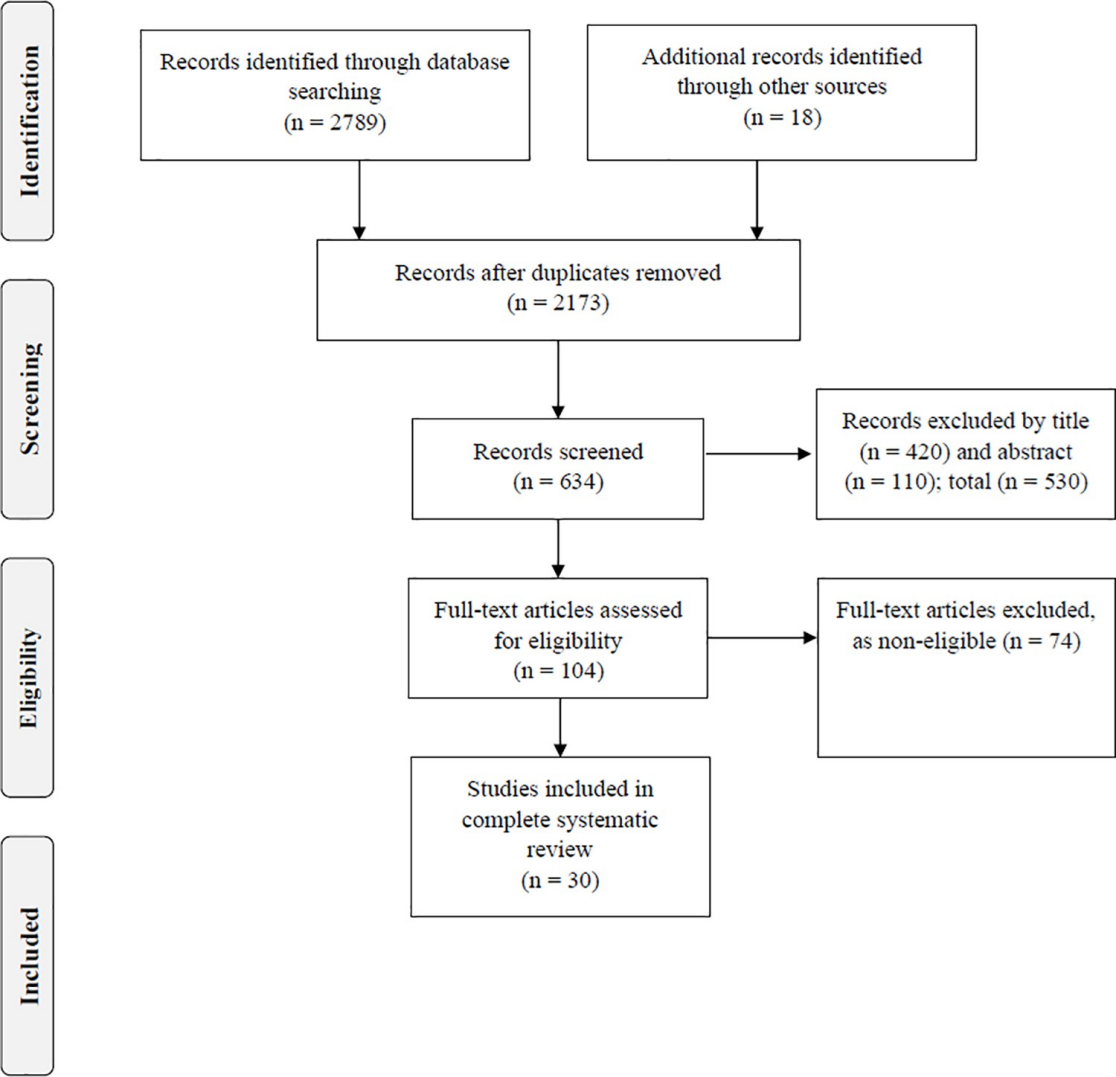
PRISMA flow diagram for the selected articles.

After exclusion of duplicates (n = 2,173), 634 articles were obtained for the screening process of the title and abstract. After reading the title and abstract, 504 records did not meet the inclusion criteria for the following reasons: other subjects (n = 388), adult population (n = 53), elderly population (n = 12), adults and elderly population (n = 10), only children (n = 8) and non-original articles (n = 19). Then, 104 full-text articles were analyzed for eligibility, and 74 were ultimately excluded. The reasons for excluding these articles included: not using LCA as a method to evaluate LCs (n = 21), not considering behavioral measures for lifestyle assessment (n = 24), analyzing other populations, such as children or adults (n = 21), lifestyle association with other risk factors not meeting the eligibility criteria for this review (n = 8), and review articles (n = 3). Finally, 30 research articles were selected for the complete systematic review (Fig 1).

In Table 2 were provides an overview of the characteristics of the 30 articles included in this systematic review. Although there was no restriction as to the year of publication, 30 studies were published after 2009. The most recent article included was published in 2021 [9]. Perhaps this is because LCA is a relatively new analysis tool to assess multivariate or latent variables, such as lifestyle. Until 2019, only one study was conducted in a middle-income country (Brazil) [20]. from 2020 onwards, 4 more articles used the LCA to assess the adolescents’ lifestyle [9–11, 13]. The other 25 articles were conducted in high-income countries. Twelve in the United States [21, 25–36], five in Finland [5, 6, 37–39], three in Australia [8, 40, 41], two in Canada [42, 43], one in Ireland [44], one in Italy [21], and one in Portugal [45]. No study was pooled (Table 2).

**Table 2:** Overview of studies included in the review.

Study	Sample characteristics						Study characteristics				Analysis Characteristics	
	Country	Population	N	Sex (N/%)	Age (years)	Race/ethnicity	Name	Design	Financing (n)	Risk of Bias^#^	Number of Criteria for LCA model	Software
Heikkala et al. [6]	Finland	Two northernmost provinces of Finland	1552	G	16 to 18 years	NR	NFBC 1986	Cohort	Yes (02)	Strong	05	MPLUS 6.11
				(871)							AIC; BIC; SSABIC; Entropy; LMR-LRT.	
				B								
				(681)								
Costa et al. [9]	Brazil	Adolescents from Florianópolis, Santa Catarina	561	G (292)	13.0 ± 1.0 year	NR	Movement study (2007)	CS	Yes (01)	Moderate	04	*poLCA packageversion1*.*41 in R* 3.6.2
				B (269)							AIC; CAIC; BIC; aBIC;	
Faria et al. [13]	Brazil	Adolescents students from IFTM, Minas Gerais	217	G (107)	16.08 ± 0.95	NR	IFTM project	CS	No	Strong	08	*poLCA package in R* 3.2.2
				B (110)							AIC; BIC; G^2^; p-G^2^; χ2; p-χ2; Entropy; Interpretability.	
Miranda et al. [11]	Brazil	Female adolescents	405	G	15.92 ± 1.27	NR	PRAEVA	CS	Yes	Strong	08	*poLCA package in R* 3.2.2
									(02)		AIC; BIC; G^2^; p-G^2^; χ2; p-χ2; Entropy; Interpretability.	
Miranda et al. [10]	Brazil	Female adolescents	405	G	15.92 ± 1.27	NR	PRAEVA	CS	Yes	Strong	08	*poLCA package in R* 3.2.2
									(02)		AIC; BIC; G^2^; p-G^2^; χ2; p-χ2; Entropy; Interpretability.	
Heikkala et al. [5]	Finland	Two northernmost provinces of Finland	1552	G	16 to 18 years	NR	NFBC 1986	Cohort	Yes (02)	Strong	05	MPLUS 6.11
				(871)							AIC; BIC; SSABIC; Entropy; LMR-LRT.	
				B								
				(681)								
Xiao et al. [36]	USA	Youth Risk Behavior Survey (2017)	14,506	G (7,834)	12–18 year	White (53.8%); Black (13.4); Hispanic (22.9%); Asian (4.3%); Other (6.0%)	YRBS (2017)	CS	No	Strong	04	STATA version 13.0
				B (6,672)							AIC; BIC; aBIC; Entropy	
Miranda et al. [20]	Brazil	Female adolescents	405	G	15.92 ± 1.27	NR	PRAEVA	CS	Yes	Strong	08	*poLCA package in R* 3.2.2
									(02)		AIC; BIC; G^2^; p-G^2^; χ2; p-χ2; Entropy; Interpretability.	
Parker et al. [40]	Australia	High school	473	G (58.6%)	14.95 ± 1.61	NR	NEArbY	CS	Yes	Strong	05	MPLUS, 7.31
									(01)			
				B (41.4%)							AIC; BIC; LMR-LRT (p); Entropy*; Class Size.	
Parker et al. [8]	Australia	High school	473	G (58.6%)	14.95 ± 1.61	NR	NEArbY	CS	Yes	Strong	05	MPLUS, 7.31
									(01)		AIC; BIC; LMR-LRT (p); Entropy*; Class Size.	
				B (41.4%)								
Balantekin et al. [25]	USA	Female adolescents	166	Girls	15	White	No	CS	Yes	Strong	NR	SAS 9.4
									(03)			
Hartz et al. [26]	USA	U.S. civilian, noninstitutionalized population	1233	G	15.4±0.7	71.2% White Non-Hispanic	NHANES	CS	Yes (01)	Strong	NR	SAS 9.4
				(48.5%)								
				B								
				(51.5%)								
Tabacchi et al. [21]	Italian	Students	883	G	16.4 ±1.4	NR	ASSO	CS	Yes	Strong	06	STATA
				(37.8%)					(01)		AIC; BIC; a-BIC; CAIC; G^2^; LL.	Plugin (PennState).
				B								
				(62.2%)								
Burdette et al. [27]	USA	School-based study of adolescents	7827	G	15.76 ±1.27	53% White	Add Health	Long.	No	Strong	02	MPLUS 7.0
				(46%)							AIC; BIC.	
				B								
				(54%)								
Lawler et al. [44]	Ireland	Secondary schools	995	G	13.72 ±1.25	71.7% White	No	CS	Yes	Strong	07	MPLUS 7.0
				(61.2%)					(02)		AIC; BIC; SSABIC; LL; Entropy; LMR-LRT; LMR-LRT (p).	
				B								
				(38.8%)								
Laxer et al. [42]	Canada	9h-12h Grade School Adolescents	18587	G	NR	73.3% White	COMPASS	CS	Yes	Strong	04	SAS 9.4
				(48.9%)					(02)		AIC; BIC; a-BIC; CAIC.	
				B								
				(51.1%)								
Evenson et al. [28]	USA	Youths	3998	G	6 to 17 years	NR	NHANES	CS	Yes	Strong	03	MPLUS 7.11
				(49.8%)					(02)		p-G^2^*; Class Size; Interpretability.	
				B								
				(50.2%)								
Kim et al. [29]	USA	9h-12h Grade School Adolescents	18253	G	NR	NR	YRBS	CS	No	Weak	01	MPLUS 7.2
				(50.5%)							Interpretability.	
				B								
				(49.5%)								
Kim et al. [30]	USA	9h-12h Grade School Adolescents	12081	G	NR	56.4% White Non-Hispanic	YRBS	CS	No	Strong	08	MPLUS 7.2
				(5972)							AIC; BIC; SSABIC; LL; 2LL; LMR-LRT (p);	
				B								
				(6109)								
											ACP; Interpretability.	
Balantekin et al. [31]	USA	Adolescents of Central Pennsylvania	166	Girls	Age 5 to age 15	White Non-Hispanic	No	Cohort	Yes	Strong	05	SAS
									(01)		AIC; BIC; G^2^; Entropy; Interpretability	9.4
Carson et al. [43]	Canada	Secondary schools	19831	G	15.7	72.8%	COMPASS	CS	Yes	Strong	04	SAS
				(51.3%)		White			(02)		AIC; BIC; a-BIC; CAIC.	9.3
				B								
				(48.7%)								
Pereira et al. [45]	Portugal	5th grade	686	G	9–11 years	NR	ISCOLE	CS	No	Strong	07	MPLUS 6
				(55.5%)							AIC; BIC; p-G^2^; χ^2^; LMR-LRT; LMR-LRT (p); BLRT.	
				B								
				(44.5%)								
Heikkala et al. [37]	Finland	Two northernmost provinces of Finland	1552	G	16 to 18 years	NR	NFBC 1986	Cohort	Yes (02)	Strong	05	MPLUS 6.11
											AIC; BIC; SSABIC; Entropy; LMR-LRT.	
				(871)								
				B								
				(681)								
Jaaskelainen et al. [38]	Finland	Two northernmost provinces of Finland	6945	G	16 years	NR	NFBC 1986	Cohort	Yes (01)	Strong	05	MPLUS 6.11
				(3598)							AIC; BIC; SSABIC; Entropy; LMR-LRT (p).	
				B								
				(3347)								
Iannotti and Wang [32]	USA	Students in grades 6h to 10h	9174	G	11 to 16 years	NR	HBSC	CS	Yes (02)	Strong	05	MPLUS 5.1
				(4659)							AIC;	
				B							BIC; SSABIC; LL; ACP.	
				(4323)								
Liu et al. [33]	USA	Adolescents	7506	G	15.4	White non-Hispanic	No	CS	Yes (01)	Strong	04	MPLUS
				(3641)							BIC; LL; Entropy; LMR-LRT (P for k–1).	
				B		G						
						60.6%						
				(3865)								
						B						
						62.4%						
Straker et al. [41]	Australia	Adolescents Offsprings of Raine study	646	G	14	NR	Raine	CS	Yes (02)	Strong	06	Latent Gold 4.5
				(350)							BIC; p-χ^2^; LL; p-p-G^2^; Entropy; Classification Error.	
				B								
				(293)								
Patnode et al. [34]	USA	Children and adolescents from 6th-11th grade	720	G	14.7±1.8	84.7%	IDEA;	CS	Yes (02)	Strong	03	SAS PROC LCA 9.1
				(51.1%)		White	ECHO				AIC; BIC; G^^2^^	
				B								
				(48.9%)								
Liu et al. [35]	USA	adolescents in grades 7h to 12h	13339	G (50.8%)	15.64(B)	White	Add Health	Long.	Yes	Strong	02	MPLUS 5.1
						B (70%)			(01)		BIC*; LMR-LRT*.	
						G (66%)						
				B (49.2%);	15.46(G)							
Lajunen et al. [39]	Finland	Cohort of 14 years and 17 years of Finnish twins	4643 (at 14); 4168 (at 17)	G (2117)	At 14 and at 17	NR	FinnTwin12	Cohort.	Yes	Strong	03	Latent Gold 4.0
				B (2079)					(04)		G^2^*; p-χ^2^*; Interpretability.	

^#^Quality Assessment Tool for Quantitative Studies

*Criterion value not reported.

N: Sample; B: Boys; G: Girls; n: Number of supported; LCA: Latent Class Analyze; NR: Not Reported; CS: cross-sectional; Long.: Longitudinal; AIC: Akaike Information Criterion; BIC: Bayesian Information Criterion; G^2^: Likelihood Ratio; p-G^2^: Likelihood Ratio Test; χ^2^: Pearson’s Chi-square Statistic (Goodness of fit); p-χ^2^: Pearson’s Chi-Squared Statistic Test; a-BIC: adjusted Bayesian Information Criterion; CAIC: Consistent Akaike Information Criterion; LL: Log-Likelihood; SSABIC: Sample Size Adjusted Bayesian Information Criterion; LMR-LRT: Lo-Mendell-Rubin adjusted Likelihood Ratio Test; LMR-LRT (p): Lo-Mendell-Rubin adjusted Likelihood Ratio Test p-value of significance; 2LL: 2 times the Log-Likelihood difference between k and k—1 class model; ACP: Average Classification Probability; BLRT: Bootstrap LRT p-value; df: degrees of freedom.

Ranging from 166 to 19,831; the mean size was 5,892, and the sample size was above 1,000 adolescents in 13 studies. Five studies [10, 11, 20, 25, 31] were with girls only, and 25 (83.33%) were with girls and boys. Seventeen articles did not report race or ethnicity, two were carried out with only white non-Hispanic adolescents, and the percentage of White and Caucasian ranged from 53% to 84.7%.

Most of the studies (n = 27) were part of a major research project. Two studies were the product of the Add Health, two of COMPASS, two of Northern Finland Birth Cohort 1986, two of NHANES, and two of Youth Risk Behavior Surveillance System. Twenty-Eight of the 30 studies were supported with funding. In terms of study design, the articles were cross-sectional (n = 22), cohort (n = 06), and longitudinal (n = 02). Regarding the Risk of Bias, almost all were rated Strong (n = 28), according to the Quality Assessment Tool for Quantitative Studies. One study was classified as Weak [29] and one was Moderate quality [9].

In addition, the kind of software used to analyze the LCA was verified. The following software was used: MPLUS (n = 16), SAS (n = 06), Latent GOLD (n = 02), Stata Plugin (Penn State) (n = 02), and poLCA package of statistical software R (n = 4).

The characteristics of manifest variables, the number of LCs identified in the model and the membership prevalence of manifest variables (γ), covariates, logistic regression estimates odds ratio (OR), 95% confidence interval (CI), and conclusion about adolescent lifestyles are presented as Supporting Information 2 (S2 Table).

The number of manifest variables used in the studies ranged from three to twenty. Most of the articles presented five manifest variables (n = 9), other studies used eight variables (n = 5), six (n = 2), ten (n = 2), and 15 (n = 2). In addition, there were studies that used four (n = 1), seven (n = 3), nine (n = 1), 11 (n = 1), 12 (n = 1), 13 (n = 1) and 20 (n = 1). The smallest number of manifest variables presented was three (n = 2).

The most common variable was related to PA level (28 studies, n = 57), followed by SB (25 studies, n = 55) and nutritional habits (19 studies, n = 50). Health status, such as average sleep hours, and addict behaviors, such as alcohol use were found in seven (n = 19) and four (n = 07) studies, respectively. Variables related to mental health (n = 04) and preventive behavior (n = 02) were found in one study each (S2 Table).

Eleven of the 30 articles analyzed estimated models stratified by sex, fitting a specific model for girls and another one for boys. Overall, these sex-stratified models showed the same number of LCs for both genders. However, Lawler et al. presented two different LCA models, six LCs for girls and five LCs for boys [44]. The number of LCs generated by the selected articles was between three and six. The highest number of LCs presented was four (n = 17), three LCs (n = 16), and five LCs (n = 06) (S2 Table).

Regarding the characteristics of adolescent’s lifestyle, on average, female adolescents (37.7%) had a higher membership prevalence to the LCs with negative characteristics, with physical inactivity and physical inactivity being the most common behaviors (Table 3). Boys, on the other hand, had the highest average of membership prevalence (28.2%) in LCs with mixed characteristics, with “unhealthy diet, physically active and healthy sedentary time being the most observed behaviors. For both sexes, the highest mean of belonging was found in the latent classes with a negative characteristic, with the low level of vigorous PA being the most common (Table 3).

**Table 3:** Membership prevalence and main characteristics of the latent classes (LC) in female and male adolescents.

	Characteristics of the Negative LC*			Characteristics of the Positive LC**			Characteristics of the Mixed LC***		
Groups of adolescents	Mean of membership prevalence	LC with highest prevalence	LC with lowest prevalence	Mean of membership prevalence	LC with highest prevalence	LC with lowest prevalence	Mean of membership prevalence	LC with highest prevalence	LC with lowest prevalence
**Female**	33.7%	77.5% -	6.7%—LC “Adolescents with obesity” [45]	19.2%	79% -	2.6% -	22.1%	49.6%	3%—LC “Moderate Physical Active and High Sedentary Behavior” [35]
		LC “Inactive and sedentary lifestyle” [20]			LC “Dancers, walkers, and joggers [33]	LC “Organized Run/Swim and Dance/Gym” [44]		LC—“Healthy diet, Unhealthy Physical Active, Healthy Sedentary Time” [26]	
**Male**	17.6%	26.8% -	4.3% -	24.6%	72.8% -	2.8% -	28.2%	62% -	4.2%—LC “Moderate Physical Active and high Sedentary Behavior” [35]
		LC “Sedentary” [37]	LC “Externalizing Behavior” [37]		LC “Basketball players and runners” [35]	LC “Leisure Active Gym” [32]		LC “Unhealthy diet and Physical Active, Healthy Sedentary Time” [26]	
**Both Sexes**	33.4%	76.8% -	6.9%	54.4%	89.46%	3.6%-	27.5%	65%	12.8%—LC “High Physical Active and High Sedentary Behavior” [29]
		LC “Low Vigorous PA” [28]	Lowest engagement in health-promoting behaviors [36]		LC—“Healthy”[9]	LC “Most Moderate-Vigorous Physical Active” [28]		LC—“Insufficiently Active, Better Diet Quality” [45]	

LC: Latent Classes.

*Negative LC–represent the classes with unhealthy behaviors

**Positive LC—represent the classes with healthy behaviors

***Mixed LC–represent the classes with healthy and unhealthy behaviors.

In general. the results presented in Table 3 showed that PA and SB, followed by nutritional habits, were the primary characteristics used to label the LCs for both sexes. Specifically, physical inactivity and sedentary lifestyles were the manifest variables with higher frequency in the negative latent classes in adolescent girls. On the other hand, physical exercises and sports were activities more commonly labeled as positive LCS.

After analyzing the characteristics of the LCs, we observed the types of covariates that were associated with the membership prevalence. Eleven studies reported associated covariates. There were 27 covariates related to sociodemographic characteristics: sex (n = 9), race/ethnicity (n = 8), demographics (n = 6), age (n = 4), and income (n = 1). In addition, other measures. types of covariates were used, such as parent/family influence (n = 9), diet/nutrition (n = 6), anthropometrics measures (n = 5), general health (n = 4), school (n = 3), mental health (n = 4), addictive behaviors (n = 2), and others (n = 2). Lastly, fifteen studies did not use covariates. The covariates were more likely associated with sex and anthropometric measures.

Some studies [13, 27, 43] have shown that female adolescents who were considered physically inactive showed a greater association with some health risk behavior. In contrast, the LCs that grouped female adolescents who performed dance and aerobic exercises, such as walking and running, showed an association with good health.

Other studies presented the association between body composition and LCs that represent the adolescents’ lifestyles. Laxer et al. [42] showed that adolescents with obesity belonging to the three LCs considered to be less healthy. Balantekin et al. [31] observed that adolescent girls with a higher BMI and more body fat had a more likely association with extreme dieters than non-dieters. In contrast, Liu et al. [33] showed that overweight boys had a more likely association with “football players” latent class and less likely to “basketball players” and “runners” latent classes. Lastly, Lajunen et al. [39] confirmed that male adolescents with weight problem had less chance to be “passive but sociable,” “active and sociable,” and “active but less sociable” than “passive and solitary”.

**Fig 2:**
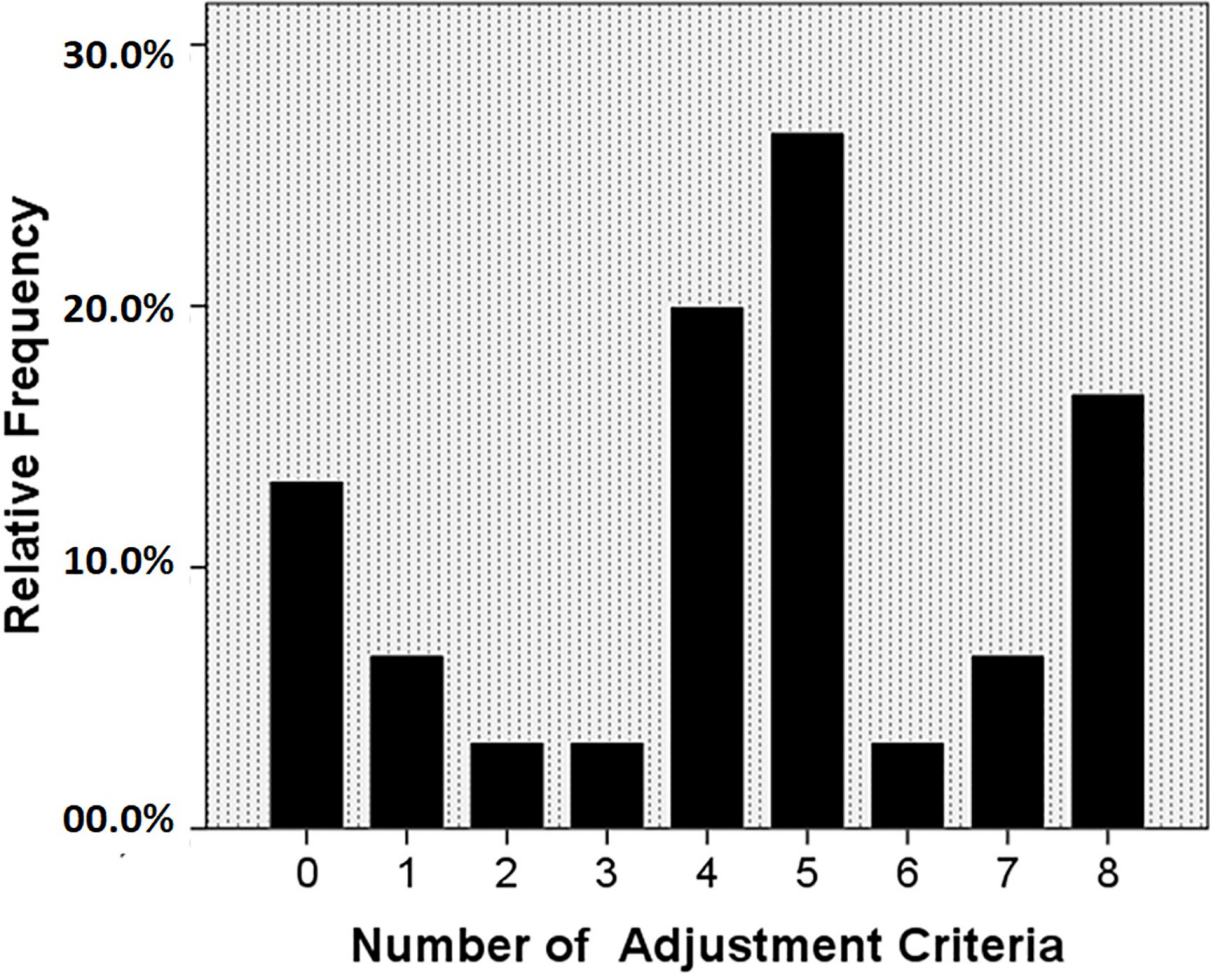
Bar chart with the number of adjustment criteria used in the Latent Class Analysis (LCA) to evaluate the adolescents’ lifestyle.

To complete our analysis of the results, we performed a descriptive analysis of the criteria cited in the articles for the selection process of the fitted model. Specifically, Fig 2 shows that the number of criteria used ranged from 0 to 8, eight (20.0%) and six (26.7%) studies used, respectively, 5 and 4 adjustment criteria. It was also observed that five articles used eight (16.7%) criteria, while four (13.3%) articles did not use any type of criterion.

The selected articles used different selection and adjustment criteria to verify for the best process of model selection. The researchers used absolute and relative model fit tests, degree of uncertainty (entropy), interpretability, and average class probabilities as strategies. All specific criteria are described in Table 4.

**Table 4:** Descriptive analysis of the criteria used by the articles (n = 30) to select the best fit LCA model.

Selection and adjustment criteria	Used Criterion n (%)	Unused Criterion n (%)	Criterion value not reported n (%)
**Relative Model Fit**			
AIC	21 (70.0)	9 (30.0)	0 (0)
BIC	24 (80.0)	5 (16.7)	1 (3.3)
LMR-LRT (p)	9 (30.0)	20 (66.7)	1 (3.3)
SSABIC	8 (26.7)	22 (76.3)	0 (0)
a-AIC	0 (0.0)	30. (0)	0 (0)
a-BIC	5 (16.7)	25 (83.3)	0 (0)
CAIC	4 (13.3)	26 (86.7)	0 (0)
LMR-LRT	2 (6.7)	28 (93.3)	1 (3.3)
BLRT	1 (4.5)	21 (95.5)	0 (0)
a-LMR (p-value for K-1)	1 (3.3)	29 (96.7)	0 (0)
**Absolute Model Fit**			
G^2^	5 (22.7)	16 (72.7)	1 (4.5)
p-G^2^	2 (9.1)	19 (86.4)	1 (4.5)
χ^2^	2 (6.7)	28 (93.3)	0 (0)
p-χ^2^	1 (3.3)	28 (93.3)	1 (3.3)
LL	10 (33.3)	20 (66.7)	0 (0)
**Degree of Uncertainty**			
Entropy	11 (36.7)	18 (60.0)	1 (3.3)
**Interpretability**	12 (40.0)	18 (60.0)	0 (0)
**Average class probabilities** *	5 (16.7)	25 (83.3)	0 (0)

n: absolute value; %: relative value; LCA: Latent Class Analysis; AIC: Akaike Information Criterion; a-AIC: adjusted Akaike Information Criterion; BIC: Bayesian Information Criterion; G^2^: Likelihood Ratio Test; p-G^2^: p-value of Likelihood Ratio Test; χ^2^: Pearson’s Chi-square Statistic; p-χ^2^: Pearson’s Chi-Squared Statistic Test; a-BIC: adjusted Bayesian Information Criterion; CAIC: Consistent Akaike Information Criterion; LL: Log-Likelihood; SSABIC: Sample Size Adjusted Bayesian Information Criterion; LMR-LRT: Lo-Mendell-Rubin adjusted Likelihood Ratio Test; LMR-LRT (p): Lo-Mendell-Rubin adjusted Likelihood Ratio Test p-value of significance; ACP: Average Classification Probability; BLRT: Bootstrap LRT p-value.

* Whitesell et al. [46].

The methods of assessment for the relative fit of competing models were presented in more depth in the articles. Mainly, the Akaike Information Criterion and Bayesian Information Criterion criteria, with 70.0% (n = 21) and 80.0% (n = 24), respectively, were the primary models used for criteria analyses of the most adjusted model. Two studies did not report the criteria used [25, 26]. Besides that, the likelihood-ratio test (G^2^), the significance of G^2^, Pearson’s chi-square test (χ^2^), the p-value of χ^2^, and log-likelihood were used to evaluate the absolute model fit.

The interpretability was cited in 12 (40.0%) papers. Thus, the investigators judged whether the model was interpretable and provided useful insights compatible with concepts of the phenomenon studied. Five (16.7%) articles used the average classification probabilities (ACPs). These ACPs showed the degree to which the classes could reliably be distinguished from one another. According to Whitesell et al, ACPs approaching or exceeding 0.80 were preferred [46].

## Discussion

This Scoping review selected 22 articles that used LCA as a method for assessing adolescent’s lifestyles. The physical inactivity, high SB were the most common in the LCs with negative characteristics of the adolescents’ lifestyle. These behaviors represented the adolescents’ lifestyle in the articles selected for this scoping review. As a highlight, the girls presented higher membership prevalence in LCs that represent physical inactivity, high SB, and the adoption of unhealthy nutritional habits [20]. It was verified that weight problems, cardiometabolic diseases, and mental health disturbances was associated with LCs of adolescents’ lifestyle. Outcomes of predominant unhealthy behavior of adolescents’ lifestyle, strengths, limitations, future studies, and practical applications were also observed.

Adolescence is an important stage in life in the development and maintenance of health-related behaviors. However, risk behaviors identified as physical inactivity, sedentary behavior, unhealthy eating habits, and the use of alcohol, drugs, and tobacco [47] are common during adolescence, especially in girls [6]. Sedentary behaviors tend to increase with age [8], increasing the prevalence of adolescents with obesity [48, 49].

In addition, it was found that psychosocial diseases can also be associated with the behavior of adolescents. As an example, inactive adolescents with high BS showed greater dissatisfaction with body image [11] and with signs of depression and anxiety [13]. However, Xiao et al. [36] showed that adolescents in the classes with adequate PAL, SB and nutritional habits, when compared to those in classes with lowest engagement in health-promoting behaviors, showed an increase in suicidal behaviors.

Most studies utilized a robust sample (over 1,000 participants). In fact, to perform LCA, Collins and Lanza recommend large sample sizes because the criteria of absolute model fit tests (ΔG^2^) has high power for detecting even minor differences in large samples [50]. Another relevant aspect was that three studies were with girls only, and other nine studies presented fitted models stratified by sex, with specific results for girls and for boys. Investigating the lifestyle habits of girls is very important. Scientific evidence argues that girls teach themselves can be more inactive and sedentary than boys [51–53]. For example, Miranda et al. developed a lifestyle assessment model specifically for Brazilian adolescent girls aged 14–19 years [20].

Nineteen were part of major projects and were supported by grants. The range of projects developed, and the research findings show that the lifestyle of adolescents is a current and relevant topic. There is no doubt that the unhealthy lifestyles of adolescents have a major impact on a country’s public health [10]. Therefore, adopting strategies to stimulate the development of projects to prevent various lifestyle-related diseases is of paramount importance. The involvement of government research funding and the development of policies will positively benefit public health, the economy, and the society of a country [54].

The articles were mostly cross-sectionally designed, as expected, due to the epidemiological and observational characteristics of the studies about lifestyle behaviors in this population [6, 50, 55]. Additionally, 28 studies were considered to have presented strong evidence, in accordance with the criteria of the risk of bias assessment [24].

PA and SB were the manifest variables most investigated in the articles on adolescent lifestyles. Nutritional habits and health status were other manifest variables observed Addict behaviors, mental health, and preventive behavior were less emphasized in the studies. The number of manifest variables ranged from three to 20, and the most common number of manifest variables was five.

In fact, recent studies suggest that lifestyle behaviors should be analyzed from a multifactorial perspective, considering different types of behaviors together [2, 20]. Even PA and SB should not be considered functional opposites or “two sides of the same coin.” [6]. Thus, the complex interplay and the underlying mechanisms between these behaviors should be considered in intervention programs with a lifestyle change approach.

Girls appear almost twice as much as the boys in negative LCs. On the other hand, boys were most prevalent in the positive LCs related to adolescent lifestyles. In studies that analyzed both genders together, the prevalence was 31.6% in negative and 21.2% in positive LCs, respectively. These results confirm what is already established in the literature on the lifestyle of adolescents. Girls have presented a lifestyle more inactive and sedentary than boys [1, 2, 6, 20]. On the other hand, physical exercises and sports were activities more commonly labeled as positive LCS. According Torstveit et al. [56], adolescents participating in organized sports had decreased odds for engaging in several unhealthy lifestyle habits compared with non-participants, indicating that organized sports may be a relevant setting for promoting healthy behaviors among adolescents [56].

Currently, adolescents spend several awake hours performing low-energy or sedentary activities, such as watching television, using smartphones, playing video games, or using the computer [9, 55, 56]. In accordance with this systematic review, other studies have shown that, for psychosocial and cultural reasons, the time girls spend on physically inactive and sedentary activities increases from adolescence to early adulthood [1].

Regarding PA, the number of adolescents worldwide that do not meet the minimum recommendation of 60 minutes of moderate or vigorous PA is about 80.3% [57], and girls were 30% more inactive than male adolescents [58]. Saunders, et al. showed that American and Canadian adolescents spend 40% to 60% of their awake daily time in a sitting position or using screen media [53]. The American Academy of Pediatrics considers screen time (ST) in front of a television, video game, or computer of <2 hours per day to be ideal [59]. However, studies show that in developed countries, approximately 70% of children and adolescents have ST above the recommended time [60, 61]. In addition, an excess ST reflects a lower daily energy expenditure, triggering risk factors for cardiometabolic diseases and impairing mental health and even academic performance [19].

Mixed LCs were also identified, with manifest variables from different types and opposite behaviors related to lifestyle, such as a healthy diet, unhealthy PA, and healthy sedentary time [41]. The average prevalence of boys was higher than that of girls in the mixed LCs. It is important to consider that in the adolescent population, the interaction between categories that represent healthy and unhealthy behaviors is common [20].

The first study that used LCA to assess adolescent lifestyles was published in 2009. Before then, researchers had used variable-centered approaches like correlation or factor analyses, which examined the relationships between isolated variables, like MVPA, SB, and diet separately. In this variable-centered approach model of analysis, intra-individual differences in the sample may be neglected [43].

In this sense, LCA is more effective in assessing the lifestyles of adolescents because it does not impose a predefined concept of what is a healthy behavior. Therefore, LCA is a more focused approach on the characteristics of the individuals, which may be homogeneous or heterogeneous, in accordance with the actual data structure [16]. An issue when using LCA is that the multiple characteristics that classify individuals into subgroups are examined simultaneously. If analyzed separately, they increase type I errors [50, 62]. In addition, LCA does not limit the number of possible classes or subgroups, and it is possible to evaluate the interaction of different types of behavior [19].

Thus, clustering techniques, such as LCA, can provide insights into patterns of health behaviors, especially those that may not seem intuitively related [42]. One explanation may include the theory of problem behavior, which suggests that an underlying behavioral syndrome causes adolescents to engage in multiple problem behaviors, possibly caused by an imbalance of risk factors in relation to protective factors in the personality and socioenvironmental domains [49]. Finally, the use of LCA reinforces the understanding that risk behaviors occur concurrently, but in diverse ways, which may justify specific prevention approaches for different risk behaviors that manifest or worsen in adolescence [20].

Through the analysis of the selected articles, it was possible to observe that the health risk behaviors of adolescents do not occur separately. Evidence suggests that adolescents have a lifestyle in which they adopt patterns of various health risk behaviors in conjunction with non-modifiable covariates, such as race or ethnicity, and modifiable covariates, such as physical inactivity and sedentary behavior [43, 63, 64]. The manifestation of weight problem, risk factors for cardiometabolic and chronic diseases [42] can be more associated with manifestation of two or more non-modifiable and modifiable covariates.

From a total of 22 studies, 11 reported covariates associated with LCs of adolescent lifestyles. Laxer et al. [42] identified that adolescents with unhealthy weight were more likely presented in the “Inactive/Screen” class than those who had a healthy weight [42]. Kim et al. verified that adolescents with low PA and high SB had less chance of sufficient sleep hours than high PA and low SB [29]. Adolescent girls that had “never consumed alcohol” were more likely to belong to the class “active and sedentary lifestyle” than to class “inactive and sedentary lifestyle”[20]. Thus, analyzing covariates associated with these health risk behaviors is fundamental to identify other factors that help explain the lifestyles adopted by adolescents. Additionally, intervention strategies for the prevention of these diseases are usually specific to single risk factors when they should be multifactorial [65].

This systematic review focused on behaviors related to the lifestyles of adolescents directly or indirectly related to body fat accumulation, weight problem and, consequently, risk factors for cardiometabolic diseases [66]. However, it is noteworthy that, currently, research has verified the association of these behaviors with body image [67], eating disorders 12, and anxiety and depression [68].

Physical inactivity and SB, combined with an unhealthy eating patterns [69], can be considered a primary factor related to obesity in childhood and adolescence [20]. Unhealthy weight is a multifactorial problem, and the energetic imbalance that occurs between habitual PA and food intake are an important factor to be considered.

The accumulation of fat, especially in the abdominal region of the body, triggers increased blood pressure and dyslipidemia [9, 70]. The condition is further aggravated by the fact that excess triglycerides and free fatty acids are important factors that cause adipocyte hypertrophy and hyperplasia [71]. Also, inflammatory markers and alteration of gut microbiota showed association with increased BMI, high BF%, elevate waist and neck circumference of female adolescents [72]. The metabolic complications may activate the release of pro-inflammatory cytokines, such as interleukin-6 (IL-6) and tumor necrosis factor α (TNF-α), and decrease the release of anti-inflammatory drugs, such as interleukin-10. IL-6 and TNF-α stimulate the production of C-reactive protein by the liver [71], and together, they trigger the subclinical inflammation process, which in turn may result in the onset of cardiovascular diseases during adolescence [20, 73].

Thus, it is evident that the assessment of lifestyle characteristics of adolescents may allow the identification of important factors involved in body fat accumulation and, consequently, the clinical manifestations of cardiometabolic diseases and also in the process of subclinical inflammation [10]. Identifying distinct patterns of health behaviors can help researchers better understand the etiological factors of overweight among adolescents and has important implications for health promotion and public health [42, 65]. The logic behind a focus on grouping stems from the recognition that the behavior of a group of people is multivariate and interactive in nature [74]. For example, PA, SB, and eating patterns may combine in a complex way and have a cumulative effect on the development of weight problem [69, 74]. These results can demonstrate which variables should be investigated simultaneously and how groupings of obesogenic behaviors can be used to assist in the development of initiatives to prevent unhealthy weight and its comorbidities. Additionally, adolescents who presented insufficient PA reported an elevated use of alcohol and tobacco, no preventive behaviors, and less self-perception of academic performance [20, 42, 75, 76].

This systematic review has some limitations. This systematic review focused on studies with adolescents in the sample. However, only in three studies [28, 31, 45], the age range included children. However, the majority in both was composed of a population aged 10 years or over, and the authors classified the sample as youths, adolescents. Only one of the selected studies was conducted in a middle-income country. All the other articles were from high-income countries. Future studies could be conducted in middle- and low-income countries, as well as focusing on cohort or longitudinal studies. We also recommend further research that considers as manifest variables other components of adolescents’ lifestyle, such as sleep habits, addictive behaviors, and mental health.

This is the first study that investigated the use of LCA to assess adolescent lifestyles through a systematic review. In addition, the results discussed also provides support for future research using LCA to access adolescent’s lifestyle. Based on the summary of these results, it was demonstrated that educators and health care professionals should be able to create and propose preventive measures and more efficient interventions that can help promote increased PA level, reduce sedentary behavior, and improve the eating habits of adolescents. Thus, reducing the incidence of children and adolescents with obesity, as well as diminishing the action of risk factors for cardiometabolic diseases and body image disorders manifested.

## Conclusions

The LCA models from the majority of selected studies showed that physical inactivity, high SB were the most common in the LCs with negative characteristics of the adolescents’ lifestyle. Girls presented higher membership prevalence in LCs that represent physical inactivity, high SB, and the adoption of unhealthy nutritional habits.

Thus, from the analysis of the most prevalent of LCs, it was evident that more comprehensive and multifactorial intervention strategies can be more effective if designed with a focus on promoting PA, reducing SB, acquiring adequate dietary habits, and adopting other behaviors related to a healthy lifestyle. Better understanding the results of analyses of clusters of multivariate behaviors such as the insights provided by LCA can help to create more effective strategies that can make the lifestyle of adolescents healthier. Because the chances of weight problems and other risk factors for cardiometabolic and psychological diseases may be lower during adolescence, diminishing the chances that they can worsen or develop into other comorbidities in adulthood is an important goal.

## Supporting information

S1 Table Prisma checklist. Moher D, Liberati A, Tetzlaff J, Altman DG, The PRISMA Group (2009). Preferred Reporting Items for Systematic Reviews and Meta-Analyses: The PRISMA Statement. PLoS Med 6(7): e1000097. doi:10.1371/journal.pmed1000097.
(DOC)

S2 Table Latent class analysis, variables, covariates, outcomes, and conclusion about lifestyle of adolescents. MV: Manifest Variables; LC: Latent Classes; PAL: Physical Activity Level; SB: Sedentary Behavior; NH: Nutritional Habits; BMI: Body Mass Index; AH: Addictive Habit; HS: Health Status; MVPA: Moderate to Vigorous Physical Activity; ST: Screen time; NR: Not Reported; NI: Not Investigated; CRP: C-reactive protein.
(DOCX)

## Abbreviations

MVPA: moderate-to-vigorous physical activity
LCA: latent class analysis
LC: latent class
PA: physical activity
SB: sedentary behavior
OR: odds ratio
CI: confidence interval
ACP: average classification probability
