# Supplementary material for: Use of latent class analysis as a method of assessing the physical activity level, sedentary behavior and nutritional habit in the adolescents’ lifestyle: A scoping review
Source: PLoS One. 2021 Aug 19;16(8):e0256069. doi: 10.1371/journal.pone.0256069 (PMC8376087; doi:10.1371/journal.pone.0256069)
Supplement: S2 Table — MV: Manifest Variables; LC: Latent Classes; PAL: Physical Activity Level; SB: Sedentary Behavior; NH: Nutritional Habits; BMI: Body Mass Index; AH: Addictive Habit; HS: Health Status; MVPA: Moderate to Vigorous Physical Activity; ST: Screen time; NR: Not Reported; NI: Not Investigated; CRP: C-reactive protein. (DOCX) [file pone.0256069.s002.docx]

**S2 Table: Latent Class Analysis, variables, covariates, outcomes, and conclusion about lifestyle of adolescents.**

| **Study** | **Number of Latent Classes identified in model (γ) and prevalence** | **Number of Manifest Variables** | **Covariates** | **Association between Covariates and LCA** | **Conclusion related to LCA** |
| --- | --- | --- | --- | --- | --- |
| Heikkala et al. [6] | Girls  04 LC  C1: Externalizing Behavior (γ=15%);  C2: Multiple Risk Behaviors (γ=11.8%);  C3: With obesity (γ=6.7%);  C4: Reference (γ=66.5%).  Boys  04 LC  C1: Externalizing Behavior (γ= 4.3%);  C2: Sedentary (γ=26.8%);  C3: With obesity (γ=7.8%);  C4: Reference (γ=51%). | 07 MV  01 PAL:  Physical activity h/day.  01 SB:  Sitting time h/day.  02 HS:  Sleeping h/day;  BMI kg/m^2^.  01 HA:  Smoking.  02 MH:  Internalizing problems;  Externalizing problems. | NI | Unhealthy behaviors and psychosocial problems are associated with later labor market exclusion, and  whether multisite musculoskeletal pain. | The Externalizing behavior cluster associated with over one year of unemployment  and permanent work disability in the follow-up among the men. The Sedentary cluster also  associated with over one and under one year of unemployment and no employment days (among the men. Obese male participants were at risk of over one year of unemployment and no employment days |
| Costa et al. [9] | 02 LC  C1: Unhealthy (10.4%)  C2: Healthy (89.6%) | 03MV  MVPA time, Screen time and Sleeping. | NI | NI | Most adolescents were grouped in the healthy class and had higher cardiorespiratory fitness levels than those in the unhealthy class |
| Faria et al. [13] | 03 LC  C1: Active & Non- Sedentary Lifestyle (γ =28.1%);  C2: Inactive & Non-sedentary Lifestyle (48.85%);  C3: Inactive & Sedentary Lifestyle (γ=23.04%). | 05MV  03PAL: MVPA time; LPA time and Number of steps measured  02 SB:  Screen Time Total (videogame, computer, tablets, smartphones, and TV);  SB. All of these measured by the accelerometer | Sex and Signs of Common Metal Disorder (CMD) | Female adolescents  presented 4.48 (95% CI 2.04–9.77) times more chance of belonging to the “Inactive-  Sedentary”. Adolescents who presented CMD had 11.35 (95% CI 3.45–101.1)  times more chance of belonging to the “Inactive—Non-sedentary” (class 2). | The interaction  between sex and signs of CMD showed that girls with signs of CMD were 9.20 (95% CI  1.17–71.52) more likely to belong to the Inactive—Sedentary class than the “Active—Non-sedentary”. |
| Miranda et al. [11] | 03 LC  C1: Active & Sedentary Lifestyle (γ=6.19%).  C2: Inactive &Non-sedentary Lifestyle (16.31%);  C1: Inactive & Sedentary Lifestyle (γ =77.5%) | 05 MV  02 PAL:  MVPA time; Number of steps.  02 SB:  Screen Time Total (videogame, computer, tablets, smartphones, and TV);  Sitting time.  01 NH:  Number of meals | Alcohol & Association with body image disorder | Never Alcohol Consumption and body image dissatisfaction had more likely association to Active and Sedentary Lifestyle instead Inactive and Sedentary Lifestyle. | Subjects with an  ‘Inactive and Sedentary’ latent lifestyle were 1·71 times as likely to feel dissatisfied  as those with active and sedentary or inactive and non-sedentary lifestyles (95 % CI  1·08, 2·90, p = 0·047). |
| Miranda et al. [10] | 03 LC  C1: Active & Sedentary Lifestyle (γ=6.19%).  C2: Inactive &Non-sedentary Lifestyle (16.31%);  C1: Inactive & Sedentary Lifestyle (γ =77.5%) | 05 MV  02 PAL:  MVPA time; Number of steps.  02 SB:  Screen Time Total (videogame, computer, tablets, smartphones, and TV);  Sitting time.  01 NH:  Number of meals | Alcohol. Association with body composition, cardiometabolic factor risk diseases and inflammatory markers. | Never Alcohol Consumption and high body fat percentage, insulin resistance and c-reactive protein had more likely association to Active and Sedentary Lifestyle instead Inactive and Sedentary Lifestyle. | “Sedentary & Inactive LS” LC together with the high levels of weight and BF% were associated with increased levels of blood pressure, lipid  profile, uric acid and  concentrations of proinflammatory biomarkers of tumor necrosis factor-α, interleukin-6, and  leptin. |
| Heikkala et al. [5] | Girls  04 LC  C1: Externalizing Behavior (γ=15%);  C2: Multiple Risk Behaviors (γ=11.8%);  C3: With obesity (γ=6.7%);  C4: Reference (γ=66.5%).  Boys  04 LC  C1: Externalizing Behavior (γ= 4.3%);  C2: Sedentary (γ=26.8%);  C3: With obesity (γ=7.8%);  C4: Reference (γ=51%). | 07 MV  01 PAL:  Physical activity h/day.  01 SB:  Sitting time h/day.  02 HS:  Sleeping h/day;  BMI kg/m^2^.  01 HA:  Smoking.  02 MH:  Internalizing problems;  Externalizing problems. | NI | The classes “Externalizing behavior”  among both genders “Multiple risk behaviors” among girls and a  “Sedentary” cluster among boys were associated to recurrent  multisite Musculoskeletal pain. | Adolescents with psychosocial difficulties and/or several adverse health behaviors were at an increased risk of recurrent multisite Musculoskeletal pain, which emphasizes the importance of simultaneously studying multiple rather than single factors. |
| Xiao et al. [49] | 04 LC  C1: Consistently Engaged in Health-Promoting  Behaviors (HPB) (γ=23%); C2: Irregular Diet, Moderate Exercise, and High Computer Use (γ=37.7%); C3: Moderate Diet, Frequent Exercise, and Moderate Sleep (γ=31.8%); C4: Lowest engagement  in health-promoting behaviors (γ=6.9%) | 13 MV  frequencies of physical activity and sports team participation.  media use (TV/  computers; Sleep; frequency of consuming breakfast, fruits/vegetables,  soda) | NI | Class Lowest engagement  in health-promoting behaviors had higher odds of suicide plan than Class 1 (OR = 1.50, 95%  CI=1.10–2.05). | Both Class 1 (consistent) and Class 4 (lowest) engagement in health-promoting behaviors were  associated with increased suicidal behaviors. |
| Miranda et al. [20] | 03 LC  C1: Inactive and Sedentary Lifestyle (γ =77.5%);  C2: Inactive and Non-sedentary Lifestyle (16.31%);  C3: Active and Sedentary Lifestyle (γ=6.19%). | 05 MV  02 PAL:  MVPA time; Number of steps.  02 SB:  Screen Time Total (videogame, computer, tablets, smartphones, and TV);  Sitting time.  01 NH:  Number of meals. | Alcohol. | Never Alcohol Consumption had more likely association to Active and Sedentary Lifestyle instead Inactive and Sedentary Lifestyle. | After PAl evaluation (MVPA), number of steps, screen time, total sitting time and number of meals and alcohol consumption (covariate) was found.  03 LC and none was active and non-sedentary.  It has been found that unhealthy lifestyle behaviors are easier to group together than healthy lifestyles. |
| Parker et al. [39] | 03 LC  C1: Physically Inactive and  Highly Sedentary (γ=44%);  C2: Moderately Active and High Screen-time (γ=42%);  C3: Highly Active and  Low Sedentary (γ=14%). | 08 MV  03 PAL:  Active travel to/from school;  Leisure-time sport or physical activity;  MVPA.  05 SB:  Watching TV;  Electronic media;  Video games;  Homework; | NI | NI | Differences between LC in: age, gender, weight status, cultural identity, employment status and sedentary time.  Low prevalence of highly active and low sedentary.  High prevalence of weight problem. |
| Parker et al. [8] | 03 LC  “Physically inactive, highly sedentary” (γ=44%); (2) “Moderately active, high screen-time” (γ=42%); and (3) “Highly active, low sedentary” (γ=14%). | 08 MV  03 PAL:  MVPA time; Active travel to/from school; Leisure-time physical activity  04 SB:  Sedentary time; TV viewing; video gaming; Electronic media.  Other:  Homework | NI | Adolescents in “Highly active, low sedentary” (class 3) tended to be younger, of higher socioeconomic position and lower BMI. | Older adolescents have less active, more sedentary profiles than younger adolescents. |
| Balantekin et al. [25] | 04 LC  C1: Non-dieters (γ=26%);  C2: Lifestyle (γ=16%);  C3: Dieters (γ=43%);  C4: Extreme Dieters (γ=15%). | 20 MV  20 NH:  Twenty weight-control behaviors from the comprehensive  list developed by French et al. (1995). | NI | NI | Variables related to family, friends, peers, and the media have an influence on the weight control of female adolescents.  Parents, media, and peers influences the adoption of diets for food control.  Family functioning and the priority of family meals serve as protection for the adoption of unhealthy methods for weight control. |
| Hartz et al.^.^[26] | 03 LC (Girls; Boys)  Girls:  C1: Healthy Diet and PA, Unhealthy ST (γ=5.7%);  C2: Healthy Diet, Unhealthy PA, Healthy ST (γ=49.6%);  C3: Unhealthy Diet, PA, and ST (γ=44.7%).  Boys:  C1: Healthy Diet and PA, Unhealthy ST (γ=10.3%);  C2: Unhealthy Diet and PA, Healthy ST (γ=62%);  C3: Healthy Diet, Unhealthy PA and ST (γ=27.8%). | 03 MV  01 PAL:  Accelerometer.  01 SB:  Time SB.  01 NH:  Diet. | NI | NI | In girls, modifiable risk factors for cardiovascular disease cluster significantly and healthy characteristics do not necessarily cluster.  In boys, latent classes showed association with factors related to cardiorespiratory fitness. |
| Tabacchi et al. [21] | 05 LC  C1: Virtuous (γ=30.7%);  C2: Low PA/Sport (γ=18.8%);  C3: Alcohol/Food Habits (γ=25.8%);  C4: Health Risk/Overweight (γ=15.9%);  C5: Malaise/Diseases  (γ=8.8%). | 11 MV  02 PAL:  Moderate PAL; Sport.  01 SB:  Screen watching.  02 NH:  Non-adequate Meals;  Food habits.  04 HS: Adolescents with obesity;  At health risk;  High malaise; Frequency  Diagnosed diseases.  02 AH:  Alcohol consumption; Smoking. | NI | NI | The lowest levels of physical fitness were found in adolescents to the "low PAL/ Sports".  The highest frequencies of low skills were found in the "Low PAL/Sport" and "Health Risk/Overweight".  The "Virtuous" and "Alcohol/Food Habits " presented lower number of subjects with low levels of fitness.  A greater risk of having all health-related fitness components low was found for the "low PAL/Sport".  Adolescents in the "Health Risk/Overweight" had a higher risk of low muscular resistance, while the "Malaise/Diseases" presented a significant increase in the risk of lower body strength, muscular endurance, speed and agility. |
| Burdette et al. [27] | 04 LC  C1: Low Risk (γ=32%);  C2: Moderate Risk with Substance Use (γ=15%);  C3: Moderate Risk with Inactivity (γ=32%);  C4: High Risk (γ=21%). | 08 MV  01 PAL:  Exercised at least 3x in  past week.  01 SB:  Watched TV or played video  games no more than 2h  per day in past week.  02 NH:  Usually eats breakfast;  Ate fruits or vegetables at least  2x yesterday.  01 HS:  Usually sleeps 8–11h per night.  02 AH:  No smoking in past 30 days;  No binge drinking in past month.  01 PB:  Always wears seat belt when  driving or riding in a car. | Age; Sex; Race; Born; Urbanicity; Region; Parent Education; Income; Parental Health; Early Health;  Parental Alcoholism; Smoker in Household. | Age had more likely association to Moderate Risk with Inactivity than Low Risk.  Female had more likely association to  Moderate Risk with Inactivity than  Low Risk.  Smoker in Household  had more likely association to High Risk than Low Risk.  African American, Foreign-born, Intact family structure  had more likely association to  Low Risk than High Risk. | The theory of healthy lifestyle is applicable to healthy behavior of the adolescent.  Four latent classes emerge during adolescence.  Although adolescents with low-risk and high-risk lifestyle, 47% of adolescents were classified as having moderate risk.  The study showed that lifestyle classified as high risk, particularly those involving substance use, compromise adolescent health in the short and long term. |
| Lawler et al. [43] | Girls:  06 LC  C1: Organized Run/Swim and  Dance/Gym (γ=2.6%);  C2: Active Individual Sport (γ=9.5%);  C3: Organized Dance (γ=12.8%);  C4: Walk/Run/Outdoor games (γ=17.1%);  C5: Leisure Active Team Sport (γ=23%);  C6: Non-Participation (γ=35%).  Boys:  05 LC  C1: Leisure Active Gym (γ=2.8%);  C2: Leisure Active Individual  Sport (γ=17.6%);  C3: Non-Participation (γ=23.1%);  C4: Active Mixed Type (γ=25.1%);  C5: Active Team Sport (γ=31.4%). | 05 MV  05 PAL:  ≥60mins  /3 days;  Non-Organized  PAL ≥ 3 sessions;  Team Sport;  Individual Sport;  Organized Non-Sport. | NI | NI | Girls classified as "Non-participation" of PAL presented the lowest scores of intrinsic motivation, competence, autonomy and relatedness with peers. |
| Laxer et al. [41] | 04 LC  C1: Traditional School Athletes (γ=24.1%);  C2: Inactive Screenagers (γ=43.3%);  C3: Health Conscious (γ=16%);  C4: Moderately Active Substance Users (γ=16.6%). | 15MV  04 PAL:  Low PAL;  Low strength;  No varsity;  No intramural.  03 SB:  High Internet;  High video game;  High TV.  05 NH:  Low Fruit and Vegetables;  Low breakfast;  High fast food;  High corner store;  High Sugar-sweetened.  03 AH:  Smoker;  Marijuana;  Binger Drink. | Adolescents with obesity. | Adolescents with obesity had more likely association to  Traditional School Athletes than Health Conscious.  Adolescents with obesity had more likely Inactive Screenagers  than Health Conscious.  Adolescents with obesity had more likely  Moderately Active Substance Users than Health Conscious. | Adolescents belonging to the three groups considered to be less healthy had a higher risk of being considered with unhealthy weight. |
| Evenson et al. [28] | Four models  Sedentary  04 LC  C1: Most Sedentary (γ=13.5%);  C2 (γ=30.1%);  C3 (γ=38.5%);  C4: Least Sedentary (γ=18.%).  Light PA  04 LC  C1: Least Light (γ=12.3%);  C2 (γ=29.3%);  C3 (γ=41.8%);  C4: Most Light (γ=16.6%).  MVPA  04 LC  C1: Least MVPA (γ=59.9%);  C2 (γ=33.3%);  C3 (γ=3.1%);  C4: Most MVPA (γ=3.6%).  Vigorous  03 LC  C1: Least Vigorous (γ=76.8%);  C2 (γ=18.5%);  C3: Most Vigorous (γ=4.7%). | 04 MV  03 PAL  Light activity;  Moderate to vigorous;  Vigorous activity.  01 SB  SB time. | NI | NI | The sample presented a mean of 50.9% SB or 6.8 h/day.  43.8% Light PAL or 349 min/day.  5.3% MVPA or 42.7 min/day.  1.6% vigorous PAL or 12.7 min/day. |
| Kim et al. [29] | 04 LC  C1: High PAL and Low SB (γ=30.5%);  C2: High PAL and High SB (γ=12.8%);  C3: Low PAL and High SB (γ=39.5%);  C4: Low PAL and Low SB (γ=17.2%). | 05 MV  03 PAL:  Regular PAL;  Sports  Team participation;  Muscle-strengthening exercise.  02 SB:  Watching television;  Playing video or computer games or using a  computer for non-school–related work. | Sleep Quantity. | Low PAL and High SB had less chance of sufficient sleep hours than High PA and Low SB.  High PA and High SB had less chance of sufficient sleep hours than High PA and Low SB. | Adolescents with high PAL and low SB were more likely to have 8 hours or more of sleep.  Boys were more prevalent in high PAL and low SB.  Girls, more advanced grade, sexual intercourse and alcohol and tobacco consumption were more prevalent in the classes with low PAL and High SB. |
| Kim et al. [30] | 04 CL (Girls; Boys)  C1: High PAL and High SB (γ=17.6%; γ=20.3%);  C2: High PAL and Low SB (γ=23.1%; γ=38.6%);  C3: Low PAL and High SB (γ=26.4%; γ=7.7 %);  C4: Low PAL and Low SB (γ=33%; γ=33.5%). | 05 MV  03 PAL:  Active sports team participation;  Sufficient moderate or vigorous physical activity;  Sufficient  muscle-strengthening exercise.  02 SB:  TV hours;  Computer hours. | Gender; Grade  (9th–12th); Race/Ethnicity. | Boys and Girls in the earlier grade and White/Non-Hispanic were less likely to belong to High PA and Low SB.  Boys and Girls in the Low PA and High SB were more chance to weight problem than Boys and Girls in High PA and Low SB. | PAL and SB are lifestyle behaviors associated with obesity.  Adolescents with obesity had high PA and low SB were more likely to comply with the current recommendations of PAL and SB, especially girls. |
| Balantekin et al. [31] | 04 LC  C1: Non-Dieters (γ=26%);  C2: Lifestyle (γ=16%);  C3: Dieters (γ=43%);  C4: Extreme Dieters (γ=15%). | 09 MV  01 PAL:  Increase exercise.  07 NH:  Skip meals;  Eat less meal;  Eat low calorie food;  Reduce calories and amount of food;  Eliminate snaking, sweets, junk;  Eat less fat;  Increase fruits/vegetables;  01 HS:  Unhealthy behavior. | BMI; Body  Fat; Restraint; Weight Concerns; Self-esteem; Depression;  Binge Eating; Dieting frequency. | BMI, Body Fat, Restraint, Weight Concerns, Depression, Binge Eating, Dieting Frequency  had more likely association to Extreme Dieters than Nondieters.  Self-esteem had less chance of association to Extreme Dieters than Nondieters. | Three dietary patterns were ordinally identified (lifestyle, diet, and extreme diet) with an increase in the number of weight-control behaviors.  Initial weight and weight control were identified as early risk factors for adherence to extreme diet. |
| Carson et al. [42] | 03 LC  C1: Healthiest Movers (γ=31%);  C2: Active Screenies (γ=25%);  C3: Unhealthiest Movers (γ=44%). | 06 MV  02 PAL:  Moderate PAL;  Hard PAL.  03 SB:  Television;  Internet;  Video games.  01 HS:  Sleep. | Age; Sex; Race/Ethnicity; Sugar-sweetened  Beverage Consumption; Fruit and Vegetable Consumption. | Female  had more likely association to Active Screenies  and Unhealthiest Movers  instead Healthiest Movers. | Participants with unhealthy movers behavior patterns were more likely to have weight problem, especially in girls. |
| Pereira et al. [44] | 02 LC  C1: Sedentary, Poorer Diet Quality (γ=35%);  C2: Insufficiently Active, Better Diet Quality (γ=65%). | 5 MV  01 PAL:  MVPA.  01 SB:  Screen Time.  02 NH:  Fruits/Vegetables;  Sugar Drinks.  01 HS:  Sleep | NI | NI | Both latent classes identified that children and adolescents were physically inactive or sedentary, with Insufficiently Active, Better Diet Quality having better eating habits.  Insufficiently Active, Better Diet Quality individuals were less likely to present weight problem. |
| Heikkala et al. [36] | Girls  04 LC  C1: Externalizing Behavior (γ=15%);  C2: Multiple Risk Behaviors (γ=11.8%);  C3: With obesity (γ=6.7%);  C4: Reference (γ=66.5%).  Boys  04 LC  C1: Externalizing Behavior (γ= 4.3%);  C2: Sedentary (γ=26.8%);  C3: With obesity (γ=7.8%);  C4: Reference (γ=51%). | 07 MV  01 PAL:  Physical activity h/day.  01 SB:  Sitting time h/day.  02 HS:  Sleeping h/day;  BMI kg/m^2^.  01 HA:  Smoking.  02 MH:  Internalizing problems;  Externalizing problems. | NI | NI | Behaviors related to health and psychosocial symptoms stratified adolescents in distinct subgroups.  Adverse lifestyle patterns (low levels of physical activity, high BMI and smoking) persisted between 16 and 18 years.  Among the girls, several unhealthy behaviors and psychosocial symptoms accumulated.  Inactive boys had more sleep problems. |
| Jaaskelainen et al. [37] | 03 LC (Girls; Boys)  C1: Unbalanced Weight Control (γ=35%; γ=26%);  C2: Adverse Habits  (γ=30%; γ=19%);  C3: Healthy Lifestyle (γ=31%; γ=47%). | 08 MV  02 PAL:  Physical activity;  Heavy exercise to control weight.  04 NH:  Stress-related eating;  Binge eating;  Strict diet to control weigh;  Eating meals with one’s family.  01 HS:  Sleeping.  01 AH:  Tobacco use. | NI | NI | Adolescents who reported feeding when under stress consumed more sweet and fatty foods, alcohol, used unhealthy weight management methods more frequently and were more prone to unhealthy health behaviors. |
| Iannotti and Wang [32] | 03 LC  C1: Healthful (γ=26.5%);  C2: Unhealthful (γ=26.4%);  C3: Typical (γ=47.2%). | 10 MV  01 PAL  Frequency PAL.  03 SB:  TV;  Game;  Computer.  06 NH:  Fruits;  Vegetables;  Sweets;  Soft Drink;  Chip;  French Fries. | Gender; Grade; Race/Ethnicity; Family Affluence  Scale. | Family Affluence  Scale had less chance to Unhealthful and  Typical than Healthful. | The different patterns in these classes suggest that relative rates of PA, SB, and consumption of fruit and vegetables may drive differences in weight status and mental health more than consumption of energy-dense snacks and drinks. |
| Liu et al. [33] | Girls:  05 LC  C1: Dancers, Walkers, and Joggers (γ=79.0%);  C2: Aerobic Exercisers (γ=6.1%);  C3: Swimmers (γ=5.6%);  C4: Volleyball Players (γ=4.9%);  C5: Soccer Players (γ=4.2%).  Boys:  05 CL  C1: Basketball Players and Runners (γ=72.8%);  C2: Football Players (γ=9.0%);  C3: Bicycle Riders (γ=7.5%);  C4: Soccer Players (γ=5.8%);  C5: Walkers (γ=4.7%). | 10 MV  10 PAL:  Basketball;  Running;  Football (B);  Bicycling;  Walking;  Weight lifting (B);  Jogging;  Swimming;  Soccer;  Dance;  Aerobics (G);  Volleyball (G). | Age; Race/Ethnicity; Overweight; Reference Person’s Education; Family Poverty Status; Health Status; Region; Urban-rural Residence, Season. | Girls with  Very Good Health Status had more likely association to  Aerobic Exercisers than Dancers, Walkers and Runners.  Boys Overweight  had more likely association to Football Players and less like to Soccer Players than  Basketball Players and Runners. | Team sports were more frequent among boys.  Almost 80% of the girls were from the dance/walk/run group.  Hispanic boys liked soccer more than white boys.  Black boys were more interested in soccer and less into cycling and walking.  Hispanic and black girls were less interested in swimming than whites.  Black was less interested in football.  With age, boys were less prone to group activities and more prone to individual activities.  For girls, age was associated with increased interest in aerobic exercise and declining interest in soccer.  Seasonality played an important role in the selection of leisure activities.  In winter girls were more prone to aerobic exercise, while in summer they were more likely to practice swimming. |
| Straker et al. [40] | 03 LC  C1: Instrumental  Computer Users’ (γ=42.6%); C2: Multi-modal  E-gamer (γ=28.8%);  C3: Computer  E-gamers (γ=28.6%). | 08 MV  08 SB:  Computer general;  Computer e-mail;  Computer graphics;  Computer internet;  Computer word;  Computer game;  Non-computer game;  TV. | NI | NI | Screen-based media use by adolescents did cluster and these clusters related differently to other activity/sedentary behaviors and physical and psychosocial health indicators. |
| Patnode et al. [34] | 03 LC (Girls; Boys)  C1: Active  (γ=18.7%; γ=42.1%);  C2: Sedentary  (γ=47.6%; γ=24.9%);  C3: Girls  Low Media/Functional Activity (γ=33.7%).  C3: Boys  Low Media/Moderate Activity (γ=33.0%). | 12 MV  06 PAL:  MVPA—weekdays;  MVPA—weekend days;  Traditional sports;  Fitness activities;  Other sports/physical activities;  Chores/work.  06 SB:  Watching TV;  Watching DVDs or videos;  Playing video or computer games;  Using the Internet/computer;  Talking/texting on the phone;  Reading/homework. | NI | NI | Given the significant concern regarding the high prevalence of adolescents with obesity among youth in this country, these findings may be very useful as part of the empirical basis in the guidance and planning of tailored and targeted interventions based on patterns of behaviors. |
| Liu et al^.^. [35] | 05 LC (Girls; Boys)  C1: Low PAL/Low SB (γ=55.7%; γ=29.9%);  C2: Moderate PAL/High SB (γ=3.0%; γ=4.2%);  C3: Moderate PAL/Low SB (γ=18.3%; γ=28.3%);  C4: High PAL/Low SB (γ=4.9%; γ=8.4%);  C5: High PAL (except skating/biking)/Low SB (γ=18.2%; γ=29.2%). | 06 MV  03 PAL:  Domestic tasks, hobbies; Skate and cycling; Sports and physical exercises.  03 SB:  Weekly screen time in hours (Watch television; Videos; Play electronic games). | Age; Gender; Ethnicity; Birth Country (United States or Other); Higher Parental Schooling; Family income; At school; Recommendations (MVPA or ST guideline). | Girls and Boys that meets  MVPA Recommendations had more association to High PA/Low SB  than Low PA/Low SB.  Girls and Boys that meets  ST guideline had more association to  Moderate PA/High SB than  Low PA/Low SB. | The sociodemographic characteristics  of each class can provide initial ideas on target populations.  Multifactorial interventions that promote physical activity and reduce screen time among adolescents are necessary. |
| Lajunen et al. [38] | 04 LC (Girls; Boys)  C1: Passive and Solitary (γ=7.65%; γ=18.71%);  C2: Passive but Sociable (γ=19.13%; γ=20.44%);  C3: Active and Sociable (γ=39.91%; γ=15.25%);  C4: Active but Less Sociable (γ=33.30%; γ=45.60%). | 15 MV  03 PAL:  Boys clubs  or scouts; Sports; Outdoor  activities.  12 SB:  TV; Video; Computer  games; Listening to  music; Board games; Musical  instrument  playing; Reading; Arts; Crafts  meeting peers; At home; At friends’  home; Away from  home | Pubertal Development; Parents’ schooling, BMI. | Boys  with obesity had less chance to  Passive but Sociable, Active and Sociable, Active but Less Sociable  than Passive and Solitary. | Leisure activities and level of socializing influence the development of  overweight among adolescents. |

Legend: MV: Manifest Variables; LC: Latent Classes; PAL: Physical Activity Level; SB: Sedentary Behavior; NH: Nutritional Habits; BMI: Body Mass Index; AH: Addictive Habit; HS: Health Status; MVPA: Moderate to Vigorous Physical Activity; ST: Screen time; NR: Not Reported; NI: Not Investigated; CRP: C-reactive protein.
